# Die Kohorte der 41.000 Langzeitüberlebenden des Deutschen Kinderkrebsregisters

**DOI:** 10.1007/s00103-022-03507-0

**Published:** 2022-03-16

**Authors:** Peter Kaatsch, Claudia Trübenbach, Melanie Kaiser, Friederike Erdmann, Claudia Spix, Desiree Grabow

**Affiliations:** Deutsches Kinderkrebsregister (DKKR), Abteilung Epidemiologie von Krebs im Kindesalter, Institut für Medizinische Biometrie, Epidemiologie und Informatik (IMBEI), Universitätsmedizin Mainz der Johannes Gutenberg-Universität Mainz, 55101 Mainz, Deutschland

**Keywords:** Krebs, Kindesalter, Langzeitfolgen, Zweittumoren, Krebsregister, Neoplasms, Childhood, Late effects, Second primary, Registry

## Abstract

**Hintergrund und Ziel:**

Ein Drittel der Langzeitüberlebenden nach Krebs im Kindes- und Jugendalter leidet unter schweren Spätfolgen (z. B. Zweittumoren, kardiale Probleme). Am Deutschen Kinderkrebsregister (DKKR) sind ca. 70.000 inzidente Erkrankungsfälle dokumentiert, von denen sich über 41.000 in Langzeitbeobachtung befinden und für Spätfolgenstudien kontaktiert werden können. Diese Kohorte wird beschrieben, die bisher mit dem DKKR durchgeführten Spätfolgenstudien werden charakterisiert, die Teilnahmebereitschaft wird analysiert.

**Methoden:**

Für die von 1980 bis 2019 mit Krebs diagnostizierten und am DKKR in der Langzeitbeobachtung befindlichen Patienten wurde die Verteilung nach Diagnose, aktuellem Alter, Beobachtungsdauer, Zahl an Zweittumoren zum Stichtag 16.07.2021 ermittelt. Berechnet wurden die Raten derer, die jeweils auf Verlaufsabfragen reagiert haben. Der Einfluss von Determinanten auf die Teilnahmebereitschaft wurde mithilfe von generalisierten Schätzgleichungen geschätzt.

**Ergebnisse:**

In der Kohorte von 41.466 kontaktierbaren Langzeitüberlebenden sind über 10 % der Betroffenen über 40 Jahre alt, bei über 40 % liegt die Erkrankung über 20 Jahre zurück. Die Teilnahmebereitschaft bei den Befragungen liegt zwischen 30 % und 60 %. Sie ist abhängig vom Alter bei Diagnose, dem Befragungsumfang, der Zahl der zuvor schon durchgeführten Befragungen. Optimal erscheint ein Abstand zwischen Kontaktierungen von mindestens 4 Jahren.

**Diskussion:**

Mit dieser einzigartigen Kohorte ist eine für Deutschland repräsentative Spätfolgenforschung möglich. Ein geeignetes Maß zu finden, wie häufig Überlebende kontaktiert werden dürfen, ist essenziell. Um nicht zu oft zu kontaktieren, sollte die Zahl der in eine Studie einzubeziehenden Betroffenen jeweils möglichst niedrig gehalten werden.

## Abstract (en)

**Background and objectives:**

One-third of childhood cancer long-term survivors suffer from severe late effects (e.g., secondary cancer and cardiovascular diseases). The German Childhood Cancer Registry (GCCR) holds approximately 70,000 registered cancer cases, of which more than 41,000 are long-term survivors, with recent follow-up and contact information that can be used for scientific studies on late effects. The characteristics of this cohort are presented, previous late effects studies with the support of the GCCR are described, and the respective participation rates are reported.

**Methods:**

For all patients who developed cancer between 1980 and 2019 and were in long-term observation at the GCCR, the distribution of diagnoses, current age, observation time, and number of secondary cancers as of 16 July 2021 was determined. The rates of patients who responded to history queries were computed. The influence of determinants on the participation rate were calculated using generalized estimating equations.

**Results:**

The cohort comprises 41,466 long-term survivors. Of these, 10% are older than 40 years and 40% had their cancer diagnosis more than 20 years ago. The participation rates range between 30 and 60% and depend on age at diagnosis, the complexity of the study, and the number of previously conducted surveys. A time interval of at least four years between two consecutive contacts seems optimal.

**Conclusions:**

Our unique cohort enables population-based research on late effects after childhood cancer. To define a sensible time interval for contacting survivors is essential. In order to ensure that survivors are not contacted too frequently, the number of survivors included in research projects should be as small as possible.

## Einleitung

An dem im Jahr 1980 gegründeten Deutschen Kinderkrebsregister (DKKR) werden entsprechend internationaler Kriterien systematisch flächendeckend Krebserkrankungen bei unter 18-Jährigen erfasst [1]. Zu den Aufgaben des DKKR gehört die zeitlich unbegrenzte Langzeitnachbeobachtung. Damit wird die Einbeziehung Betroffener in Spätfolgenstudien ermöglicht und vielfältig genutzt. Dies hat einen hohen Stellenwert, da mit den verbesserten Heilungschancen die Zahl derer deutlich gestiegen ist, die an potenziellen Spätfolgen leiden. So hat sich in den letzten Jahrzehnten die Zahl der länger als 5 Jahre nach Diagnosestellung lebenden („Langzeitüberlebende“) europaweit auf etwa 80 % deutlich verbessert [2].

Spätfolgen können vielfältig sein: Organschädigungen (zum Beispiel Herz, Nieren oder Gehör), Fertilitätsstörungen, körperliche Behinderungen, kognitive Leistungsminderung, verminderte Lebensqualität in Bereichen des psychosozialen Lebens, Auftreten von Folgekrebserkrankungen. Bereits im Jahr 2006 hat Kevin Oeffinger in einer viel beachteten Veröffentlichung Größenordnungen der häufigsten Spätfolgen nach Krebstherapie im Kindesalter beschrieben [3]: Bei 3034 Erkrankten zeigte sich im Vergleich zu deren 10.397 Geschwistern ein 10- bis 15fach erhöhtes relatives Risiko jeweils für Herzinsuffizienz, koronare Herzerkrankungen, Folgekrebserkrankungen und kognitive Dysfunktionen und ein 5‑ bis 10fach erhöhtes relatives Risiko für Schlaganfall, Nierenversagen, Hör- und Sehverlust. Insgesamt weisen etwa 2 Drittel der Langzeitüberlebenden Spätfolgen auf; insgesamt ein Drittel leidet unter schweren Spätfolgen [4, 5]. Häufigste Todesursachen nach Krebserkrankung im Kindes- und Jugendalter sind maligne Folgekrebserkrankungen und therapiebedingte kardiale Probleme [6–8].

In der vorliegenden Publikation wird die am DKKR existierende Kohorte der mittlerweile über 41.000 Langzeitüberlenden beschrieben, deren Erkrankung zwischen 1980 und 2019 aufgetreten ist. Dies ist eine Aktualisierung der Beschreibung aus dem Jahr 2012 mit damals ca. 25.000 Betroffenen [9]. Es werden die damalige Krebsdiagnose, die Beobachtungsdauer, das zum Stichtag aktuelle Alter, die Zahl der seit Diagnosestellung vergangenen Jahre und der Anteil der zwischenzeitlich aufgetretenen Folgekrebserkrankungen dargestellt. Darüber hinaus sind die bisher mit dem DKKR durchgeführten Spätfolgenstudien kurz charakterisiert und die damit verbundene Teilnahmebereitschaft der mittlerweile überwiegend deutlich im Erwachsenenalter befindlichen ehemaligen Patienten beschrieben.

## Methoden

### Das Deutsche Kinderkrebsregister

Das im Jahr 1980 gegründete, an der Universitätsmedizin Mainz angesiedelte DKKR ist ein epidemiologisches Krebsregister und zählt mit derzeit ca. 70.000 dokumentierten Erkrankungsfällen bei unter 18-Jährigen (bis 2008 nur unter 15-Jährige) zu den größten Kinderkrebsregistern weltweit [10]. Es wird durch die Gesundheitsministerien von Bund und Ländern finanziert und basiert auf der Einwilligungsregelung, d. h., die Eltern/Sorgeberechtigten oder bei entsprechender Einsichtsfähigkeit die Betroffenen selbst müssen ihre Einwilligung zur Datenübermittlung von der behandelnden Klinik an das DKKR geben.

Zu den klassischen Aufgaben gehören u. a. die Berechnung von Inzidenzraten, die Ermittlung zeitlicher Veränderungen oder regionaler Unterschiede sowie die Berechnung von (Langzeit‑)Überlebenswahrscheinlichkeiten. Anhand der Daten des DKKR werden epidemiologische Forschungsprojekte, z. B. zur Ursachenforschung oder zu Spätfolgen durchgeführt. Die Kooperation mit den Krebsregistern der Länder und das Einbringen deutscher Daten in internationale Verbundprojekte gehören ebenfalls zu den Aufgaben. Meldende Stellen sind die behandelnden kinderonkologischen Zentren; 3 Viertel der Erkrankungen werden derzeit aus den 28 größten Behandlungszentren gemeldet. Zwischen den behandelnden Kliniken, dem DKKR und den in der Gesellschaft für Pädiatrische Onkologie und Hämatologie (GPOH) organisierten klinischen Studien und klinischen Registern besteht ein enger Informationsverbund [11].

Jährlich erkranken etwa 2200 Personen neu vor ihrem 18. Geburtstag an malignen Erkrankungen, wobei in diesem Wert – entsprechend der International Classification of Childhood Cancer [12] – auch benigne Tumoren des zentralen Nervensystems enthalten sind.

Seit dem Jahr 2009 gehört – basierend auf einem Beschluss der Gesundheitsministerkonferenz – auch die systematische, zeitlich unbefristete Langzeitnachbeobachtung der Betroffenen zu den definierten Aufgaben des DKKR. In diesem Rahmen werden die Langzeitüberlebenden – die ältesten sind mittlerweile über 50 Jahre alt – grundsätzlich mindestens alle 5 Jahre vom DKKR kontaktiert. Darüber hinaus können die Betroffenen auch im Rahmen zusätzlicher Spätfolgenstudien um entsprechende Studienteilnahme gebeten werden. Dies können Befragungen zu bestimmten Lebensumständen oder die Teilnahme an klinischen, beispielsweise kardiologischen Untersuchungen sein.

Die hier präsentierten Ergebnisse zur Gesamtkohorte der Langzeitüberlebenden umfassen Patienten, deren Krebserkrankung zwischen 1980 und 2019 diagnostiziert wurde. Es erfolgt eine deskriptive Darstellung von Anzahl und Anteil der Patienten an der Gesamtkohorte unter Berücksichtigung von damaliger Diagnose, aktuellem Alter und Länge der Beobachtungsdauer. Es wurden alle Verlaufsinformationen herangezogen, die bis zum 16.07.2021 vorlagen.

### Die Regeln der kinderonkologischen Fachgesellschaft GPOH zur Kontaktierung Betroffener

Die GPOH hat im Laufe der Jahre in Zusammenarbeit mit dem DKKR Regeln vereinbart, damit Betroffene nicht zu oft, nicht unkoordiniert und nicht von beliebig vielen unterschiedlichen Stellen kontaktiert werden. Diese Regeln wurden in Positionspapieren veröffentlicht [13, 14]. Im Wesentlichen beinhalten sie folgende Festlegungen: Alle Vorhaben, die mit der Kontaktierung von betroffenen Langzeitüberlebenden einhergehen, müssen vorab von einem von der GPOH eingesetzten Forschungsausschuss befürwortet werden (Tab. 1). Die Kontaktierung aller Betroffenen, die nicht mehr über die seinerzeit behandelnde Klinik erreichbar sind, erfolgt ausschließlich über das DKKR.

**Table 1:** 

Regeln und Maßnahmen	Ziel
Installation des Forschungsausschusses der Gesellschaft für Pädiatrische Onkologie und Hämatologie (GPOH)	Beurteilung der Relevanz der von Dritten geplanten Vorhaben mit Kontaktierung Betroffener
	Unterstützende Koordinierung des DKKR bei miteinander konkurrierenden Vorhaben Dritter
Zentralisierte Funktionen des DKKR	Kontaktierung Betroffener ausschließlich über das DKKR
	Wohnadressenrecherche nur am DKKR
	Zentral vorliegende Information über alle bisherigen Kontaktierungen und Studienteilnahmen der Betroffenen
	Vermeidung von unkoordinierten (Mehrfach‑)Kontaktierungen
Persönliche Erstkontaktierung im Jahr des 16. Geburtstages (über die Sorgeberechtigten)	Klärung, ob die Jugendlichen über deren frühere Erkrankung und die Datenspeicherung am DKKR informiert sind
	Erstkontaktierung solange die Betroffenen üblicherweise noch bei den Eltern wohnen
	Vorbereitung späterer Kontaktierungen durch das DKKR
Regelmäßige Kontaktierung der Betroffenen	Kenntnis über neu aufgetretene Folgekrebserkrankungen und Spätrezidive (mit entsprechender Validierung)
	Hinweise, inwieweit grundsätzlich Bereitschaft zur weiteren Kontaktierung besteht
Meldeamtsrecherchen	Aktualisierung der Wohnadressen
	Information über neu aufgetretene Sterbefälle

Dazu werden am DKKR grundsätzlich die Wohnadressen und der Vitalstatus der Betroffenen in regelmäßigen Abständen über Melderegister aktualisiert. Am DKKR als zentraler Stelle liegen auch die Informationen vor, im Rahmen welcher Vorhaben Betroffene in zurückliegenden Jahren bereits kontaktiert wurden und teilgenommen haben. Grundsätzlich wird bei allen Befragungen das Auftreten von Spätrezidiven oder Folgekrebserkrankungen erfragt und dokumentiert.

### „Pflege“ der Langzeitüberlebendenkohorte

Um die immer größer werdende Kohorte der Langzeitüberlebenden am DKKR zu „pflegen“ (d. h. sie fortzuschreiben, regelmäßige Kontaktierungen zu ermöglichen, dem Informationsbedürfnis der Betroffenen und deren Informationsrecht nachzukommen), gibt es am DKKR verschiedene Maßnahmen und Regeln (Tab. 1). Im Wesentlichen sind es die Folgenden:

#### Erstmalige Kontaktierung der Betroffenen im Jugendalter.

Jedes Jahr wird festgestellt, welche Patienten im aktuellen Kalenderjahr das 16. Lebensjahr vollenden. An die Eltern dieser Patienten wird ein Schreiben mit der Bitte geschickt, dem Sohn/der Tochter einen ebenfalls beiliegenden, für die Eltern einsehbaren Umschlag auszuhändigen. In den darin enthaltenen Unterlagen stellt sich das DKKR dem Jugendlichen vor, informiert über die bestehende Datenspeicherung und darüber, dass das DKKR beabsichtigt, von Zeit zu Zeit die Betroffenen nach ihrem Gesundheitszustand zu fragen. Es bleibt den Eltern überlassen, ob sie diese Informationen („Kontaktbogen KB16“) an ihr Kind weitergeben. Es kommt gelegentlich vor, dass die betroffenen Jugendlichen nicht darüber informiert sind, dass sie an Krebs erkrankt waren. In diesem Fall könnte eine direkte, unvermittelte Kontaktierung eines jungen Erwachsenen durch ein Krebsregister problematische Folgen für die Betroffenen haben.

#### Regelmäßige Statusabfragen bei den Betroffenen.

Spätestens alle 5 Jahre erfolgt die Versendung eines 2‑seitigen Fragebogens an die Betroffenen. Hierbei wird lediglich nach möglichen Folgekrebserkrankungen oder Rezidiven und nach einer Arztadresse gefragt, über die das DKKR dann ein gegebenenfalls genanntes Ereignis validiert [9]. Durch diese Statusabfragen wird ein regelmäßiger Kontakt zu den Patienten gewährleistet. Dabei können generell auch nachsorgerelevante Informationen mit verschickt werden. Diese Kontaktaufnahme erfolgt jedes Jahr bei etwa einem Fünftel der Langzeitüberlebendenkohorte.

#### Melderegisterauskünfte.

Um die Betroffenen kontaktieren zu können, müssen der aktuelle Vitalstatus der Person und ihre aktuelle Wohnadresse vorliegen. Die Recherchen hierzu erfolgen grundsätzlich über Melderegister. Während in einem ursprünglichen Verfahren diese Recherchen erst unmittelbar vor geplanten Briefversendungen erfolgten, werden diese mittlerweile regelmäßig durchgeführt. Damit werden Adressen aktueller vorgehalten und Briefe kommen weitaus seltener als „unbekannt verzogen“ zurück. Bisher erfolgten jährlich zwischen 5000 und 10.000 Adressrecherchen. Ab dem Jahr 2022 werden es aufgrund diverser Verfahrensänderungen jährlich über 25.000 Recherchen sein. So wird für wissenschaftliche Zwecke zukünftig auch die Adresshistorie registriert.

### Vorgehensweise bei Anfragen externer zur Kontaktierung Betroffener durch das DKKR

Interessierte potenzielle Kooperationspartner, die mithilfe des DKKR Betroffene im Rahmen eines Forschungsvorhabens kontaktieren möchten, müssen dieses Vorhaben zunächst dem von der GPOH eingesetzten „Forschungsausschuss Langzeitfolgen“ vorlegen und es befürworten lassen [15]. Bei der Bewertung spielt auch eine Rolle, dass es ggf. „konkurrierende“ Vorhaben gibt, bei denen eine Überschneidung der zu kontaktierenden Individuen vorliegen kann. Ohne Befürwortung durch den Forschungsausschuss schreibt das DKKR keine Betroffenen an. Die am DKKR entstehenden Kosten für die Durchführung der Befragung sind vom Antragsteller mitzufinanzieren, der hierfür zugrunde gelegte Kostenrahmen ist mit der GPOH abgestimmt.

Das „Standardverfahren“ ist dann Folgendes: Das DKKR verschickt die Unterlagen der Vorhabenleitung an die Betroffenen mit dem Hinweis, dass es das entsprechende Vorhaben unterstützt. Die Betroffenen schicken den ausgefüllten vorhabenspezifischen Fragebogen, ihre Einwilligungserklärung und ggf. weitere für die Studiendurchführung erforderliche Unterlagen an das DKKR zurück. Von dort werden die Unterlagen pseudonymisiert an die Vorhabenleitung zur weiteren Verwendung weitergeleitet. Durch die Rücksendung der Unterlagen von den Betroffenen an das DKKR – und nicht direkt an die Vorhabenleitung – kann im DKKR das Responseverhalten ermittelt werden und Erinnerungen können erfolgen. Abweichungen von diesem Verfahren können abgesprochen werden.

### Analyse der Teilnahmebereitschaft

Um die Teilnahmebereitschaft zu beschreiben, wird grundsätzlich angegeben, ob die Betroffenen auf die entsprechende Kontaktierung reagiert haben („Reaktionsrate“), was in der überwiegenden Zahl der Fälle einer aktiven Teilnahme an der jeweiligen Befragung/Studie entspricht. Möglicherweise entsprechen diese Angaben nicht unbedingt den letztlich aus den spezifische Spätfolgenstudien publizierten Fallzahlen, da dort teilweise zusätzliche Ein- und Ausschlusskriterien zugrunde gelegt sind.

Im Rahmen einer Masterarbeit [16] war zusätzlich die Teilnahmebereitschaft der Betroffenen bei 6 im Zeitraum von 2008 bis 2016 durchgeführten Routinestatusabfragen sowie bei 6 im gleichen Zeitraum erfolgten Spätfolgenstudien analysiert worden. Diese Analyse umfasste nicht die gesamte Kohorte Langzeitüberlebender, sondern ausschließlich ehemalige Patienten, die zum Zeitpunkt der Kontaktierung mindestens 21 Jahre alt waren. Der älteste ehemalige Patient war 48 Jahre alt. Um den Einfluss verschiedener Determinanten auf die Teilnahmebereitschaft zu untersuchen und dabei zu berücksichtigen, dass einzelne Personen in mehrere dieser Studien oder Befragungen eingeschlossen waren, wurden generalisierte Schätzgleichungen (Generalized Estimating Equations) angewendet [17].

## Ergebnisse

**Figure 1:**
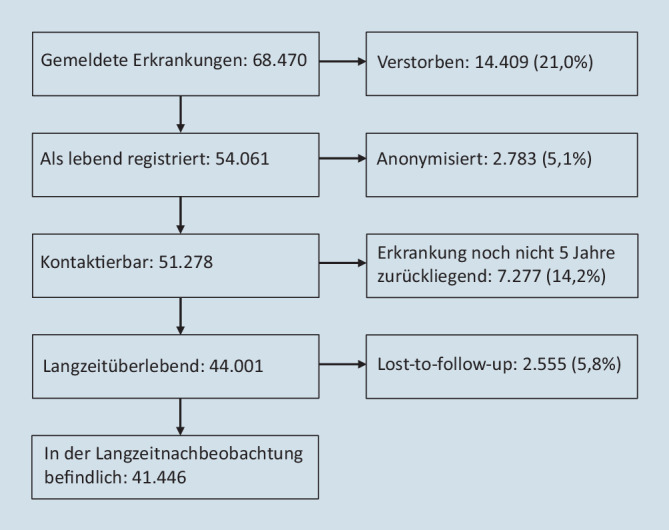

Zwischen 1980 und 2019 sind 68.470 inzidente Krebserkrankungen im Kindes- und Jugendalter an das DKKR gemeldet worden (Abb. 1). Davon sind 21,0 % als verstorben registriert. Von den übrigen 54.061 haben 2783 keine Zustimmung zur namentlichen Speicherung gegeben oder diese seither zurückgezogen, sodass eine Nachbeobachtung nicht möglich ist. Bei weiteren 7277 Betroffenen liegt die Erkrankung weniger als 5 Jahre zurück und damit werden sie hier nicht zu den Langzeitüberlebenden gezählt. Von den verbleibenden 44.001 Betroffenen sind 2555 aus dem Follow-up ausgeschieden (z. B. keine aktuelle Adresse recherchierbar). Somit befinden sich am DKKR zum genannten Stichtag 41.446 der bis 2019 gemeldeten Patienten in der aktiven Langzeitnachbeobachtung.

Details zur Beschreibung der Kohorte sind in Tab. 2 wiedergegeben: Die mediane Beobachtungsdauer liegt bei 16 Jahren 3 Monaten. Das mediane Alter zum Stichtag der Auswertung beträgt 25 Jahre 2 Monate; 10,2 % der Betroffenen sind mindestens 40 Jahre alt. Bei 43,4 % liegt die Erstdiagnose mindestens 20 Jahre zurück, davon bei 6459 Betroffenen mehr als 30 Jahre.

**Table 2:** 

	*N*	Relativer Anteil (%)
*Gesamt*	41.446	100
*Mediane Beobachtungsdauer: *16 J 3 M		
*Aktuelles Alter (2021; in Jahren)*		
Median: 25 J 2 M		
< 20	12.289	29,6
20–29	15.486	37,4
30–39	9462	22,8
≥ 40	4209	10,2
*Patienten mit Folgekrebserkrankungen*	1033	2,5
*Jahre seit Diagnose der Ersterkrankung*		
5–9	9125	22,0
10–14	7603	18,3
15–19	6712	16,2
20–24	6294	15,2
25–29	5253	12,7
30–34	3398	8,2
≥ 35	3061	7,4
*Aktualität der Information*		
< 5 Jahre zurückliegend	38.756	93,5
≥ 5 Jahre zurückliegend	2690	6,5
*Kontakt über*		
Sorgeberechtigte	9997	24,1
Patienten	31.449	75,9
*Diagnosegruppen (sortiert nach ICCC-3)*		
Leukämien	14.645	35,4
Lymphome	6224	15,0
ZNS-Tumoren	7518	18,1
Periphere Nervenzelltumoren	2696	6,5
Retinoblastome	1087	2,6
Nierentumoren	2719	6,6
Lebertumoren	424	1,0
Knochentumoren	1683	4,1
Weichteilsarkome	2131	5,1
Keimzelltumoren	1639	4,0
Karzinome	638	1,5
Andere und unspezifizierte	42	0,1

*N* Anzahl, *Mediane* angegeben in Jahren (J) und Monaten (M), *ICCC‑3* International Classification of Childhood Cancer, Third Edition [12], *ZNS* Zentrales Nervensystem

Bei 93,5 % der 41.446 Betroffenen liegen am DKKR aktuelle Informationen aus den letzten 5 Jahren vor. Bei 75,9 % sind die erwachsenen Betroffenen direkt Ansprechpartner, bei den übrigen erfolgt der Kontakt noch über die Sorgeberechtigten; dieser Anteil deckt sich in etwa damit, dass 23,1 % der Betroffenen zum Stichtag noch unter 18 Jahre alt waren.

Häufigste Krebsdiagnosen sind in der Kohorte der Langzeitüberlebenden Leukämien (35,4 %), Tumoren des zentralen Nervensystems (18,1 %) und Lymphome (15,0 %; Tab. 2). Für Leukämien und Lymphome entspricht dieser relative Anteil etwa dem Anteil dieser Diagnosen an den insgesamt an das DKKR gemeldeten Krebserkrankungen. Hingegen sind die Tumoren des zentralen Nervensystems (23,6 % aller insgesamt gemeldeten Erkrankungsfälle) infolge der generell schlechteren Prognose in der Kohorte der Langzeitüberlebenden unterrepräsentiert. 2,5 % der derzeit noch lebenden Langzeitüberlebenden weisen eine Folgekrebserkrankung auf. Dies ist niedriger als die aus dem DKKR berichtete kumulative Inzidenz nach 35 Jahren von 8,0 % für das Auftreten von Folgekrebserkrankungen [7], da ein größerer Anteil Betroffener anlässlich ihrer Folgekrebserkrankung verstorben und somit nicht in der hier beschriebenen Kohorte enthalten sein kann.

Tab. 3 zeigt die Reaktionsraten bei den beiden Routineaktionen seit dem Jahr 2009, d. h. bei der ersten Kontaktaufnahme im Jahr des 16. Geburtstages („Kontaktbogen KB16“) sowie bei den regelmäßigen „Statusabfragen“. Angegeben sind die Anzahl der postalisch erreichten Adressaten sowie die Anzahl und der Anteil der Patientenreaktionen. Bei den KB16-Aktionen (derzeit durchschnittlich etwa 1400 Kontaktierungen/Jahr) liegt der Anteil von reagierenden Jugendlichen bzw. deren Eltern zwischen 68 % und 77 %, eine relevante Veränderung über die Zeit ist nicht zu erkennen. Bei den sich für jeden Betroffenen in der Regel alle 5 Jahre wiederholenden Statusabfragen (jährlich durchschnittlich etwa 3600 Kontaktierungen) ist über die Zeit ein Rückgang der Teilnahmebereitschaft von über 60 % in den Jahren 2009/2010 bis zu 30 % im Jahr 2017 festzustellen. Hierbei spielte eine über die Jahre zu verzeichnende Häufung der Kontaktierungen durch zusätzliche Befragungen in diversen Spätfolgenstudien eine Rolle. Für die Jahre 2018 und 2019 war die Strategie des Vorgehens jeweils geändert worden, was mit 14 % und 62 % zu sehr unterschiedlichen Raten führte. Dabei ergab sich die niedrige Rate aus einer Sonderaktion zu Betroffenen, die schon mehrfach nicht reagiert hatten; die höhere Teilnahmebereitschaft für das Jahr 2019 ergab sich, weil dabei der Anteil derer besonders hoch war, deren letzter Kontakt zeitlich weit zurücklag.

**Table 3:** 

	KB16			Status		
Jahr	Erreicht	Reagiert (absolut/relativ)		Erreicht	Reagiert (absolut/relativ)	
2009	3383	2532	75 %	10.101	7018	69 %
2010	1431	1028	72 %	463	311	67 %
2011	1294	991	77 %	3690	1979	54 %
2012	1787	1280	72 %	2196	1208	55 %
2013	1784	1236	69 %	2913	1680	58 %
2014	1521	1061	70 %	2631	1145	44 %
2015	1488	1064	72 %	3099	1362	44 %
2016	1160	833	72 %	4599	1713	37 %
2017	1514	1032	68 %	4808	1427	30 %
2018	1230	872	71 %	2403 ^a^	338	14 %
2019	1567	1065	68 %	2928 ^a^	1818	62 %
2020	1523	1070	70 %	–	–	–

*„KB16“* erste Kontaktaufnahme durch das DKKR im Jahr des 16. Geburtstages, *„Status“* Versendung eines zweiseitigen Fragebogens zu Folgekrebserkrankungen, Rezidiven u. a.

^a^ Während im Jahr 2018 ausnahmsweise zu einem relativ großen Teil diejenigen angeschrieben wurden, die im Jahr vorher bzw. schon mehrfach nicht reagierten, wurden 2019 vorwiegend Betroffene angeschrieben, deren letzte Kontaktierung bereits mehrere Jahre zurücklag

Seit 2008 wurden in Zusammenarbeit mit externen Kooperationspartnern für insgesamt 12 größere Studien Langzeitüberlebende über das DKKR kontaktiert. Diese Studien mit teilweise sehr unterschiedlichem Fokus sind in Tab. 4 charakterisiert. Es handelte sich diagnoseübergreifend z. B. um Fragestellungen zu psychosozialen Aspekten [18], Fertilitätsstörungen [19], Folgen für die Nachkommen Betroffener [20], Lebensqualität [21], kardiologischen Spätfolgen [8], genetischen Assoziationen bei Folgekrebserkrankungen [22] oder schulischen Problemen [23]. Diagnosespezifische Spätfolgenstudien betrafen Morbus Hodgkin und die Langerhans-Zell-Histiozytose (LCH). Der Anteil der auf die Kontaktierung Reagierenden lag zwischen 35 % und 68 %, bezogen jeweils auf die bei der Kontaktierung postalisch erreichten potenziellen Teilnehmer. Die beiden niedrigsten Raten waren bei 2 Studien zur Kardiotoxizität zu verzeichnen, die für die Studienteilnehmer mit besonders hohem Aufwand verbunden waren, da sie zu einer kardiologischen Untersuchung eigens anreisen mussten.

**Table 4:** 

Studienkurzbezeichnung	Studieninhalt	Projekthauptverantwortliche/r	Zeitraum der Kontaktierung (Monat/Jahr)	Reaktionsrate		
				Erreicht	Reagiert	Relativer Anteil
–	Psychosoziale Adaption langzeitüberlebender onkologischer Patienten nach Erkrankung in der Adoleszenz	Lutz Goldbeck; Ulm	01/2008–12/2008	1875	905	48 %
FeCt	Fertilität nach Chemo- und Strahlentherapie im Kindes- und Jugendalter	Anja Borgmann-Staudt; Berlin	07/2008–12/2008	4673	2967	63 %
VIVE	Basiserhebung zu Lebenssituation, Gesundheitszustand und Lebensqualität nach onkologischer Erkrankung im Kindes- und Jugendalter	Gabriele Calaminus, Katja Baust; Bonn	02/2014–04/2015	10.323	5793	56 %
Nachkommenstudie	Studie zum Gesundheitszustand der Nachkommen ehemaliger onkologischer Patienten	Anja Borgmann-Staudt; Berlin	04/2013–11/2016	1304	875	67 %
CVSS	Cardiac and vascular late sequelae in long-term survivors of childhood cancer	Jörg Faber, Hiltrud Merzenich, Philipp Wild; Mainz	09/2013–01/2016	2894	1002	35 %
KiKme	Krebserkrankung im Kindesalter und molekulare Epidemiologie	Manuela Marron; Bremen	04/2016–06/2018	1766	760	43 %
ikidS-OEVA	Onkologische Erkrankung im Vorschulalter und der Übergang in die Schule	Michael Urschitz; Mainz	07/2016–09/2017	513	351	68 %
Kardiotox	Ursache und Früherkennung der Anthrazyklin-induzierten Kardiomyopathie bei Kindern und Jugendlichen nach Behandlung eines Nephro- oder Neuroblastoms	Thorsten Langer; Lübeck	06/2017–09/2018	2172	825	38 %
InfoOnko	Evaluation der psychosozialen Situation von Langzeitüberlebenden einer Krebserkrankung im Kindes- oder Jugendalter	Rebecca Toenne, Anika Mohr; Hannover	11/2017–09/2018	1884	1162	62 %
E‑SURV	Entwicklung innovativer Strategien zu Datenerhebung, Datenaustausch und Follow-Up nach Krebs im Kindesalter und Verknüpfung epidemiologischer und klinischer Daten	Gabriele Calaminus, Katja Baust; Bonn	04/2018–09/2018	1966	936	48 %
LCH2018	Basiserhebung zu Lebenssituation, Gesundheitszustand und Lebensqualität nach Erkrankung an Langerhanszell-Histiozytose	Milen Minkov, Elfriede Thiem; Wien	08/2018–11/2018	92	60	65 %
LEaHL	Spätfolgen nach Hodgkin-Lymphom	Ulrike Hennewig, Dieter Körholz; Gießen	10/2018–08/2019	1263	732	58 %

Anzahl der postalisch erreichten Betroffenen („*erreicht*“) und der dazugehörigen positiven Rückmeldungen („*reagiert*“)

Die Analyse der in der genannten Masterarbeit [16] eingeschlossenen 6 Routinestatusabfragen und 6 Spätfolgenstudien (KiKme, Kardiotox, InfoOnko, E‑SURV, LCH2018, LEaHL; Tab. 4) ergab Folgendes: Im genannten Zeitraum wurden insgesamt 5268 Betroffene einmal angeschrieben, 3490 je 2‑mal, 3403 je 3‑mal, 1797 je 4‑mal und 186 wurden im Rahmen dieser Befragungen 5‑mal kontaktiert. Diese 12 Aktionen umfassten somit insgesamt 30.575 Anschreiben an 14.144 Betroffene mit einer Gesamtreaktionsrate von 54 %.

Hierbei erwies sich der Abstand zwischen 2 Befragungen als relevantes Kriterium in Bezug auf die Teilnahmebereitschaft: Während die durchschnittliche Reaktionsrate bei 2‑jährigem Abstand zwischen 2 Befragungen bei 42,0 % lag (bezogen auf 1721 Anschreiben) und bei 3‑jährigem Abstand bei 28,4 % (2504), stieg sie bei 4‑ und 5‑jährigem Abstand auf 43,5 % (1031) und 56,6 % (4969) an. Darüber hinaus waren Einflüsse festzustellen von Geschlecht, dem Alter zum Zeitpunkt der damaligen Diagnosestellung, dem Umfang der Befragung, dem Antwortverhalten bei der erstmaligen Kontaktierung im 16. Lebensjahr, der Anzahl der bereits zuvor durchgeführten Befragungen und auch dem Vorliegen einer Folgekrebserkrankung. Tab. 5 zeigt die entsprechenden relativen Risiken.

**Table 5:** 

Einfluss auf die Teilnahmebereitschaft	RR	KI
Frauen gegenüber Männern	1,22	1,19–1,25
Bei Diagnose um 5 Jahre älter	1,04	1,02–1,05
Geringerer Umfang der Befragung	0,77	0,75–0,78
Bei aktiver Antwort auf Kontaktbogen („KB16“)	2,33	2,08–2,60
Rückgang pro zusätzlicher Befragung	0,91	0,90–0,92
Im Fall einer aufgetretenen Folgekrebserkrankung	1,30	1,24–1,38

Im Detail zeigte sich Folgendes: Frauen antworteten zu 22 % häufiger als Männer. Die Teilnahmerate stieg mit dem Alter bei Diagnose kontinuierlich an (pro 5 Altersjahre um 4 %); so antworteten bei Diagnosestellung 14-jährige Patienten um 9 % häufiger als die bereits im ersten Lebensjahr Erkrankten. Ein kurzer Fragebogen hat eine bessere Chance, beantwortet zu werden, als ein langer Fragebogen. So nahmen bei einem Fragebogen von mehr als 4 Seiten die Betroffenen mit 23 %iger Wahrscheinlichkeit weniger oft teil als bei einem kürzeren Fragebogen; bei Studien, die über eine reine Befragung hinausgingen (z. B. Einladung zur körperlichen Untersuchung), war die Teilnahmebereitschaft noch niedriger. Bei Betroffenen, die bereits aktiv bei der Kontaktierung im Alter von 16 Jahren geantwortet hatten, war die Teilnahmerate bei anschließenden Kontaktierungen mehr als doppelt so hoch gegenüber denen, die initial nicht reagiert hatten. Pro zusätzlicher Befragung nahm die Reaktionsrate um ca. 9 % ab. Patienten mit einer Folgekrebserkrankung nahmen zu 30 % häufiger teil als Patienten ohne Folgekrebserkrankung. Das Alter der Betroffenen bei Kontaktierung, die seinerzeit gestellte Diagnose oder das Auftreten eines Rezidivs zeigten keinen Einfluss auf die Teilnahmebereitschaft.

## Diskussion

Die Kohorte von 41.466 Langzeitüberlebenden, deren Krebsdiagnose länger als 5 Jahre zurückliegt und die am DKKR als lebend und weiterhin kontaktierbar registriert sind, ist in dieser Form einzigartig. Mehr als 10 % dieser Betroffenen sind bereits 40 Jahre und älter, bei über 40 % liegt die Erkrankung länger als 20 Jahre zurück, das mediane Alter dieser Kohorte beträgt 25 Jahre. Das DKKR erhält jährlich etwa 2200 Meldungen neu erkrankter Kinder und Jugendlicher. Bei einer Überlebenswahrscheinlichkeit von über 80 % und berücksichtigend, dass nicht alle Patienten nachbeobachtet werden können, kommen jährlich deutlich über 1000 potenzielle Langzeitüberlebende hinzu.

Durch die namensbezogene Speicherung ist es grundsätzlich möglich, zeitlich unbefristet von diesen ehemaligen Patienten mit Krebs im Kindes- und Jugendalter Basisdaten zum allgemeinen Gesundheitszustand zentral vorzuhalten und die Betroffenen für die aktive Teilnahme an der Spätfolgenforschung zu gewinnen. Da das DKKR ein epidemiologisches Krebsregister mit hoher Vollzähligkeit ist, ist mit dieser Kohorte grundsätzlich eine bevölkerungsbezogene, d. h. für Deutschland repräsentative Forschung, möglich. Diese Repräsentativität der Langzeitüberlebendenkohorte ist aufgrund der erfreulich wenigen aus dem Follow-up herausgefallenen Betroffenen und des geringen Anteils anonymer Erkrankungsfälle (zusammen lediglich 10,9 %) kaum eingeschränkt, auch wenn nicht ganz ausgeschlossen werden kann, dass für bestimmte wissenschaftliche Fragestellungen – auch durch die jeweilige Nichtteilnahme – gewisse Selektionen gegeben sein können. Insgesamt ist die Vollständigkeit der Kohorte auch im internationalen Vergleich außergewöhnlich hoch.

In Deutschland ist dank der geschaffenen Strukturen eine gute Basis für die Durchführung von Spätfolgenstudien nach Krebserkrankung im Kindes- und Jugendalter gegeben, zumal es neben dem DKKR noch weitere etablierte, sich mit Spätfolgen befassende Gruppen gibt. Auch der GPOH-Forschungsausschuss Langzeitfolgen sowie die GPOH-Arbeitsgemeinschaft Langzeitbeobachtung sind in diesem Kontext als wichtige Bestandteile der etablierten Struktur zu nennen [14].

Trotz der nennenswerten Gesamtzahl von derzeit über 41.000 kontaktierbaren Langzeitüberlebenden in Deutschland ist es insbesondere für spezielle Fragestellungen angeraten, Spätfolgenforschung auf internationaler Ebene durchzuführen, um ausreichend große Fallzahlen für eine hohe Aussagefähigkeit entsprechender Studien zur Verfügung zu haben.

Hierzu sei auf eine Übersichtsarbeit über die in Europa bestehenden Kohorten nach Krebsbehandlung im Kindesalter [24] ebenso verwiesen wie auf 4 innerhalb des europäischen PanCare-Netzwerkes [25] initiierte europäische Studien: PanCareSurFup (Laufzeit: 2011–2017) hat sich mit Zweittumoren, kardialen Spätfolgen und Spätmortalität beschäftigt [26, 27], PanCareLIFE (2013–2018) mit genetischen Determinanten von Fertilitätsproblemen und Schwerhörigkeit nach platinhaltiger Chemotherapie sowie mit Lebensqualitätseinschränkungen [28, 29]. In der PanCareFollowUp-Studie (seit 2019) werden, basierend auf bereits existenten Nachsorgeempfehlungen, personenzentrierte Nachsorgemodelle in 4 europäischen Ländern implementiert [30]. Forschungsgegenstand in PanCareSurPass (seit 2021) ist, den bereits entwickelten digitalen Pass für Krebsüberlebende („Survivorship-Passport“) in Europa semiautomatisch und zudem breiter als bisher zu implementieren [31]. Diese PanCare-Projekte beziehen sich grundsätzlich auf Betroffene, die im Kindes- oder Jugendalter an Krebs erkrankten.

Der Datenschutz ist besonders sensibel zu handhaben. Daher findet die Erstkontaktierung der Betroffenen grundsätzlich durch das DKKR statt und die Rücksendung der Befragungsunterlagen erfolgt in der Regel auch an das DKKR. Dort werden die Fragebögen und ggf. weitere Unterlagen in einer pseudonymisierten Form an die Vorhabenleitung zur dortigen Datenerfassung, Auswertung und Publikation geschickt. Falls aus organisatorischen Gründen (z. B. bei notwendiger Terminierung von Untersuchungen) von diesem Verfahren abgewichen werden muss, wird dies durch entsprechend ergänzte Einwilligungserklärungen, gegebenenfalls Datenschutzkonzepte und Kooperationsvereinbarungen klar geregelt. Bisher erfolgen die vom DKKR durchgeführten Kontaktierungen von Betroffenen noch auf konventionellem postalischen Weg, da solche Versendungen ein weniger datenschutzkritisches Potenzial haben als Befragungen etwa über Internetportale. Dennoch wird – auch im Hinblick auf die immer größer werdende Kohorte – an einer entsprechenden internetbasierten, datenschutzrechtlich sicheren Lösung gearbeitet.

Eine ethische Frage ist, inwieweit es verantwortbar ist, Überlebende nach einer schweren Erkrankung, wie es eine Krebserkrankung im Kindes- und Jugendalter darstellt, immer wieder zu kontaktieren. Dies häufiger als einmal jährlich zu tun, erscheint weder angemessen noch zielführend. Der weitaus größte Teil äußert sich hierzu nicht spontan. Manchmal teilen Betroffene mit, dass sie die Kontaktierung begrüßen („endlich kümmert sich jemand um mich“) und nur vereinzelt bitten sie darum, nicht mehr auf ihre damalige Erkrankung angesprochen zu werden. Bei der Bereitschaft, an Befragungen und spezifischen Studien teilzunehmen, spielt es jedoch eine Rolle, ob nur ein kurzer oder ein in die Tiefe gehender Fragebogen ausgefüllt werden soll, ob die Teilnahme mit einer Einladung zu einer klinischen Untersuchung oder sogar mit der Bitte verbunden ist, körpereigenes Material zur Verfügung zu stellen (z. B. Speichelprobe oder gar invasiv, wie etwa eine Hautstanze). Unsere Auswertungen haben erwartungsgemäß gezeigt, dass zu häufiges Kontaktieren die Teilnahmebereitschaft senkt. Hier den angemessenen Mittelweg im Sinne der Betroffenen einerseits und im Sinne der Beantwortung vielfältiger wissenschaftlicher Fragestellungen andererseits zu finden, ist eine Herausforderung. Unsere Ergebnisse lassen einen Abstand von mindestens 4 Jahren zwischen einzelnen Befragungen im Hinblick auf die Teilnahmebereitschaft als optimal erscheinen. Auch die von uns vorgenommene Erstkontaktierung im Alter von etwa 16 Jahren wirkt sich positiv auf die Teilnahmebereitschaft aus.

Für die praktische Arbeit am DKKR hat es sich als sehr erschwerend gezeigt, wenn Patienten in mehrere Spätfolgenstudien kurz hintereinander einbezogen werden. Hier war nicht immer das Verständnis der Projektverantwortlichen dafür gegeben, dass eine Studie erst einmal zurückgestellt wurde, weil die relevanten Patienten kurz zuvor in einer anderen Studie kontaktiert worden waren. Eine etwas weniger großzügige Festlegung der Einschlusskriterien und damit eine Reduktion der einzubeziehenden Patientenzahl verringert die Wahrscheinlichkeit, dass Patienten in mehrere Studien einbezogen werden, und ist auch im Sinne der Datensparsamkeit erforderlich.

Spätfolgen standen in früheren Jahren nicht so sehr im Mittelpunkt, wie es inzwischen der Fall ist. So war der Therapieerfolg im Sinne eines möglichst langen (ereignisfreien) Überlebens lange Zeit zentraler Inhalt der Studienfragestellungen. Mittlerweile gibt es einige klinischen Studien bzw. Therapieoptimierungsstudien (GPOH-Studien), bei denen bereits im therapiefokussierten Studienprotokoll spätfolgenspezifische Fragestellungen enthalten sind. Damit steht die Regel, dass grundsätzlich nur das DKKR Kontaktierungen durchführen soll, teilweise im Widerspruch zur gewünschten Vorgehensweise in diesen Studien, die gerne selbst Betroffene kontaktieren würden.

Seit jeher besteht ein enger Informationsverbund zwischen dem DKKR und den GPOH-Studien, in die über 90 % der erkrankten Kinder und Jugendlichen eingebunden sind. Diese Kooperation zwischen Register und Kliniken und der datenschutzkonforme Austausch relevanter Informationen untereinander sind mittlerweile auch im Kontext der Langzeitnachbeobachtung wichtiger Bestandteil der Arbeit des DKKR. Dies ist nicht zuletzt auch im Sinne der derzeit Betroffenen und künftig Erkrankenden von hoher Relevanz.

## Fazit

Dank der Patienten und deren Familien konnte am Deutschen Kinderkrebsregister (DKKR) eine Kohorte Langzeitüberlebender nach Krebs im Kindes- und Jugendalter aufgebaut werden. Zu über 40.000 Langzeitüberlebenden hat das DKKR regelmäßigen Kontakt. Die Betroffenen stehen grundsätzlich für die Einbeziehung in Spätfolgenstudien, z. B. zu Fertilitätsstörungen oder kardialen Problemen, zur Verfügung. Mehr als 10 derartige, für Deutschland repräsentative Studien wurden bereits durchgeführt. Die Teilnahmebereitschaft variiert und ist höher, wenn die Betroffenen weniger häufig befragt werden.
